# Urban sensory conditions alter rival interactions and mate choice in urban and forest túngara frogs

**DOI:** 10.1093/beheco/arae088

**Published:** 2024-10-26

**Authors:** Judith A H Smit, Vera Thijssen, Andrew D Cronin, Jacintha Ellers, Wouter Halfwerk

**Affiliations:** Amsterdam Institute for Life and Environment, Ecology and Evolution, Vrije Universiteit Amsterdam, De Boelelaan 1108, 1081 HZ Amsterdam, The Netherlands; Smithsonian Tropical Research Institute, Apartado 0843-03092, Balboa, Ancón, Republic of Panamá; Amsterdam Institute for Life and Environment, Ecology and Evolution, Vrije Universiteit Amsterdam, De Boelelaan 1108, 1081 HZ Amsterdam, The Netherlands; Groningen Institute for Evolutionary Life Sciences, University of Groningen, Nijenborgh 7, 9747 AG Groningen, The Netherlands; Amsterdam Institute for Life and Environment, Ecology and Evolution, Vrije Universiteit Amsterdam, De Boelelaan 1108, 1081 HZ Amsterdam, The Netherlands; Smithsonian Tropical Research Institute, Apartado 0843-03092, Balboa, Ancón, Republic of Panamá; Amsterdam Institute for Life and Environment, Ecology and Evolution, Vrije Universiteit Amsterdam, De Boelelaan 1108, 1081 HZ Amsterdam, The Netherlands; Amsterdam Institute for Life and Environment, Ecology and Evolution, Vrije Universiteit Amsterdam, De Boelelaan 1108, 1081 HZ Amsterdam, The Netherlands

**Keywords:** anthropogenic noise, artificial light at night, mate choice, rival interactions, sexual communication, urbanization

## Abstract

Sexual communication often takes place in networks with multiple competing signalers being simultaneously assessed by mate choosers. Altered sensory conditions, such as noise and light pollution, can affect communication by altering signal production and perception. While evidence of sensory pollution affecting sexual signaling is widespread, few studies assess impacts on sexual signaling during rival interactions as well as mate choice, let alone whether urban and non-urban populations have diverged in their response. Here, we investigate the effects of urban sensory conditions on sexual communication in urban and forest túngara frogs (*Engystomops pustulosus*). We recorded dyadic vocal rival interactions and assessed mate choice with and without noise and light pollution in the lab. We show that urban sensory conditions can directly impact the intensity of rival interactions, differences between rivals, and mate choice, though changes were often in opposite directions for frogs of urban and forest origins. Moreover, we demonstrate that urban-induced changes in rival interactions can also indirectly affect how females choose between potential mates. Our study reveals origin-dependent direct and indirect effects of noise and light pollution and suggests local adaptation of sexual communication in urban populations.

## Teaser

City noise and light can affect sexual communication. We show that urban sensory pollutants alter rival interactions and mate choice in túngara frogs. Interestingly, frogs from urban and forest populations often responded in opposite ways when exposed to noise and light pollution. Behavioral changes in urban populations suggest that both signalers and receivers might have locally adapted to urban conditions.

## Background

Sexual communication encompasses the production of and responses to sexual signals, such as song, pheromones, or visual displays, which have evolved in response to rival competition and mate choice (Darwin 1871; Andersson 1994). Most sexual signaling occurs in networks with multiple rivals and mate choosers, such as bird leks or frog choruses, characterized by spatiotemporal overlap in sexual signals (McGregor and Peake 2000; Gerhardt and Huber 2002). Specifically in the acoustic domain, signalers can often flexibly alter the timing and characteristics of sexual signals depending on their perception of nearby rivals (Otter et al. 2002; Greenfield 2005; Neelon and Höbel 2019; Larter et al. 2022), shaping temporal patterns and differences between signaling rivals (Greenfield 2015). Mate choosers evaluate rival interactions, and exert mate choice based on comparison between sexual signals (Mennill et al. 2002; Bateson and Healy 2005; Callander et al. 2013), often in a nonlinear and irrational manner (Akre and Johnsen 2014; Lea and Ryan 2015). Because both sexual signaling and mate choice are shaped by phenotypes of conspecifics, considering the social environment is crucial for understanding sexual communication.

How sexual signalers and intended receivers, such as rivals and mates, interact strongly depends on the sensory environment because sensory conditions can affect the production, transmission, and perception of sexual signals (Wiley and Richards 1978; Brumm and Slabbekoorn 2005). This is particularly apparent in urban environments, where anthropogenic noise and artificial light at night (ALAN) are widespread (Barber et al. 2010; Kyba et al. 2017) and have been shown to impact sensory systems of many organisms (Halfwerk and Slabbekoorn 2015; Dominoni et al. 2020). Although animals can often alter their behavior in response to urban sensory conditions, either via plastic or evolutionary responses (Lowry et al. 2013; Sol et al. 2013), it is poorly understood how urban-induced behavioral changes in signalers and receivers influence sexual communication and ultimately fitness.

In the case of signalers, direct changes in the production of sexual signals (see Cronin et al. 2022a for an extensive overview) can occur, for example, in response to noisy conditions by increasing the pitch or amplitude of their vocalizations (Cynx et al. 1998; Slabbekoorn and Peet 2003; Halfwerk et al. 2011; Lampe et al. 2012; Kunc et al. 2022), presumably to counter masking effects of noise (Halfwerk and Slabbekoorn 2015; Dominoni et al. 2020). Similarly, in response to light pollution, animals can adjust their signal composition or timing (Kempenaers et al. 2010; van Geffen et al. 2015a). Moreover, anthropogenic noise can affect how an individual perceives and responds to a rival (Luther and Magnotti 2014; LaZerte et al. 2017; Phillips and Derryberry 2018), which presumably translates into altered rival interactions (e.g. changed temporal patterns or differences between signaling rivals) with potential consequences for mate choice. For example, rivals might attempt to match each other’s signals, thereby reducing the ability of mate choosers to discriminate and express a preference. Most studies on signal adjustments under urban sensory conditions, however, take communication networks out of the equation by, for example, testing individuals in isolation or by using static playback designs (e.g. Smit et al. 2022). While such designs provide useful insights into direct effects of sensory pollutants on signalers under standardized social environments, they fail to assess how interactions between rivals and, consequently, mate choice are affected.

Urban sensory conditions can also directly impact mate choice (Candolin and Wong 2019), though the effects of sensory pollutants on mate detectability, discrimination, and preference are far from clear (Halfwerk and Slabbekoorn 2015; Dominoni et al. 2020). Noise and light pollution generally seem to decrease mate attraction toward a sexual signal (Bird and Parker 2014; Huet des Aunay et al. 2014; van Geffen et al. 2015b), most likely as a result of masking or misleading mechanisms (Halfwerk and Slabbekoorn 2015; Dominoni et al. 2020). Importantly, mating decisions are generally based on comparisons between sexual signals (Mennill et al. 2002; Bateson and Healy 2005; Callander et al. 2013), and sensory conditions can also affect how mate choosers evaluate and choose from multiple signals (Rand et al. 1997; Coss et al. 2020; Taylor et al. 2021). It is still poorly understood, however, how urban sensory conditions directly, as well as indirectly via altered rival interactions, affect mate choice between multiple signalers. Moreover, it remains to be clarified whether rivals and mate choosers from urban populations have changed their behaviors in response to urban sensory conditions.

The túngara frog (*Engystomops pustulosus*), commonly found in urban and forest areas, is an ideal study species to investigate the effects of altered sensory conditions on sexual communication in interactive settings because males aggregate in choruses and compete using acoustic sexual signals on which females base their mating decisions (Ryan 1980, 1985). Calling behavior is strongly shaped by the social environment, as males increase their call complexity (by adding “chucks,” short high amplitude elements, to their calls), amplitude, and call rate when their social environment becomes more competitive (Rand and Ryan 1981; Green 1990; Bernal et al. 2007; Halfwerk et al. 2016). Females generally prefer more conspicuous sexual signals (Rand and Ryan 1981; Bernal et al. 2009), and their preference is further shaped by rival differences and temporal dynamics (Akre et al. 2011; Tárano 2015; Larter and Ryan 2024). Frogs in urban areas produce more conspicuous calls than frogs in forest areas (Halfwerk et al. 2019), but not in the lab when using conspecific playbacks (Smit et al. 2022). Differences in the field seem partially related to urban sensory conditions (Cronin et al. 2022b; Smit et al. 2022). It is currently unclear, however, what the effects of sensory pollution are on how rivals interact and how females choose among them.

Our aim was to investigate how urban sensory conditions (noise and light pollution) impact rival interactions and mate choice, and whether the effects differ between urban and forest populations. We collected frogs from urban and forest field sites and recorded vocal interactions of urban–urban and forest–forest rival pairs under urban and forest sensory conditions in our lab set-up. Subsequently, we played the recorded dyadic (between two rivals) vocal interactions to females and quantified mate choice in phonotaxis experiments under urban or forest sensory conditions. For males, we predicted that rival interactions would become more intense under noise and light pollution, since we know that more conspicuous sexual signaling in túngara frogs is elicited by urban sensory conditions (Cronin et al. 2022b; Smit et al. 2022). Moreover, we predicted urban males to show a stronger response to urban sensory conditions, since stronger behavioral as well as physiological responses to sensory pollutants have been shown in urban túngara frog males (Halfwerk et al. 2019; Smit et al. 2024). Regarding mate choice, we predicted that urban sensory conditions would decrease female preference, because of the potentially masking, distracting, and misleading effects of noise and light pollution (Halfwerk and Slabbekoorn 2015; Dominoni et al. 2020), but we did not predict urban and forest females to differ in their responses to urban sensory conditions because this had not been shown previously (Cronin et al. 2022a). Last, we predicted that altered rival differences in response to urban sensory pollution could have indirect consequences for mate choice, with increasing rival differences resulting in higher female preference strength.

## Methods

### Study species and collection sites

We studied túngara frogs (*Engystomopos pustulosus*) from collection sites in the town of Gamboa and in Soberanía National Park, Republic of Panamá. We collected calling male frogs (to record male–male interactions) and amplectant male–female pairs (to assess preference of the females) respectively 0 to 1 and 1 to 3 h after sunset from 6 urban and 4 forest sites that were minimally 190 m apart (Fig. S1, Table S1). Urban sites were characterized by the occasional presence of humans, human-built structures, and light pollution (mean ± SD, 1.28 ± 1.10 lx, lux meter, HT Instruments HT309, see Table S1 for details), whereas forest sites were located in forested areas free from artificial light at night (< 0.01 lx). We transported frogs in small plastic containers in a plastic cooler to facilities of the Smithsonian Tropical Research Institute (STRI) in Gamboa. We recorded male–male interactions between 19:00 and 2:30 in September 2022 and conducted phonotaxis experiments assessing female preference between 20:30 and 4:00 in November 2022. We tested frogs in different batches, with male–female pairs being released at the capture site on the same night and males 3 d after capture. In total, we tested 120 males (forest: 48, urban: 72), of which 31 rival pairs (forest: 14, urban: 17) consisting of 41 males (forest: 17, urban: 24) interacted in both urban and forest sensory conditions and passed our criteria (see below). In the phonotaxis experiment, we tested 87 females (forest: 34, urban: 53, three were recaptured and retested), of which 80 females (forest: 31, urban: 49) made a total of 462 choices (forest: 170, urban: 292) out of 649 trials (see Table S1 for details). After testing, we took mass (g), body size (SVL, mm), and ventral and dorsal photographs of all frogs to be able to identify recaptures.

### Sensory treatments

**Figure 1.**
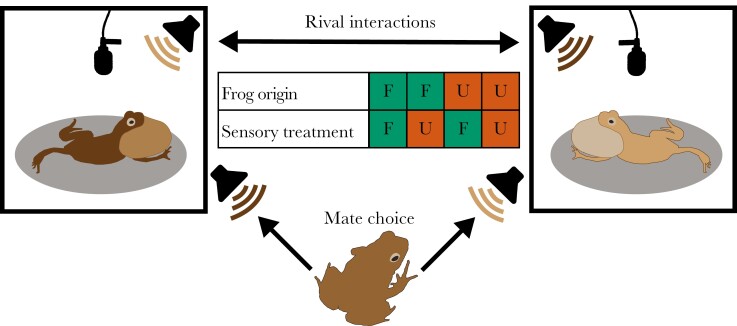
Schematic of experimental design. We recorded vocal interactions of rival pairs (urban–urban males or forest–forest males) that could hear and respond to each other in acoustically connected recording boxes. The audio recordings of the rival interactions were later played to females in phonotaxis experiments to record mate choice. We used a full factorial design regarding frog origin and sensory treatments. We conducted the experiments with rival pairs and females from urban and forest origins. During the experiments, we exposed the frogs to urban or forest sensory treatments by manipulating light and noise levels in the recording boxes and phonotaxis chamber. F and U indicate, respectively, forest and urban origins and treatments.

We exposed urban and forest males and females to urban (urban noise and light) or forest (forest noise and light) sensory conditions using a full factorial design (Fig. 1). We played synthesized forest (~50 dBA) or urban (~70 dBA) noise (peak amplitude at frog, Voltcraft SL-100, fast, low, max; see Smit et al. 2022 and Cronin et al. 2022b for details) using a speaker (Visaton FR8WP, frequency response 100 Hz to 20 kHz (−10 dB), connected to Renkforce T21 amplifiers) at ~0.1 m from the male or 2 speakers at ~1.7 m above the surface where females could move (Fig. S2). To ensure males and females would be exposed to similar noise levels, we calibrated the speakers daily using an artificial chorus playback (males: set to 67 ± 2 dBA at 20 cm, females: set to 73 ± 2 dBA at ~1.7 m; Voltcraft SL-100, fast, low, and max, Table S2). Urban noise playbacks were mostly lower frequencies (< 2 kHz), whereas the “whine” part of the túngara frog call ranges from ~ 0.4 to 1 kHz and the energy in the “chuck” is concentrated > 2 kHz, indicating some spectral overlap between the urban noise and the lowest frequency part of the call (for power spectra, see Smit et al. 2022). To manipulate light conditions, we used white broad-spectrum LEDs (Nichia NSPW500DS, peak ~460 nm) mimicking forest (< 0.01 lx) or urban light levels (~1.5 lx position of the male, ~1.95 lx starting position of the female, HT instruments HT309, Fig. S2, Table S2).

### Recording male–male interactions

We recorded vocal interactions of urban–urban and forest–forest rival pairs in a set-up with 4 sound-attenuating recording boxes (L × W × H: 36 × 25 × 58 cm) under both forest and urban sensory conditions (Fig. 1). Our set-up allowed males to hear and respond to rivals in real time by recording their calls using omnidirectional lavalier microphones (AKG C417, 40 cm above the male) connected to a 4-channel audio interface (Tascam US 4 × 4 HR or US 4 × 4) and live broadcasting the calls using the speakers that also played noise. Prior to testing each day, we played a 1.5 kHz tone (UE roll 2 Bluetooth speaker) in each recording box at the position of the frog and adjusted the microphone gain in this box so the tone would be equally broadcast (61±1 dBA at 20 cm, Voltcraft SL-100, fast, low) in all connected boxes.

Upon arrival in the lab, males were placed in tanks (AltDesign, L × W × H: 18.4 × 29.2 × 12.7 cm) with damp paper towels and wild termites ad libitum. The next day each male was transferred into a small plastic container (9 cm ø, 5.5 cm height) containing mesh on the sides for the male to hold onto while calling, a pebble as shelter and saran wrap as an acoustically permeable lid. We filled the containers with ~2.5 cm of dechlorinated water at least 0.5 h before the start of an experimental round using low level red light to reduce disturbance. Each round, males from the same collection site were randomly placed in the recording boxes (temperature 24 to 26 °C) without using light, where they were stimulated with a ~67 dB artificial chorus for 0.5 h (6 × 3 min blocks, with 2 min breaks in between) while acclimating to the first sensory treatment. Next, we turned on all microphones and waited for 2 frogs to start calling before disconnecting microphones of the other 2 males. The 2 noninteracting males were not recorded and broadcast, but they were able to hear the calls of the 2 interacting males. After at least 10 min in which the rival pair could interact, all 4 microphones were turned on again until another pair of frogs started calling. We ran 2 set-ups of 4 acoustically connected recording boxes simultaneously, and switched containers with frogs between the 2 groups of connected recording boxes, without using light, to maximize the number of different rival pairs (28 possible combinations). This procedure was repeated for 1 to 2 h until all frogs that seemed motivated to call had had the chance to interact with each other, and we started with the next group of males. We recorded 3 groups of eight males per night, which we repeated in the same order the following day in the second sensory treatment. In total, we repeated the procedures for 5 cohorts of 24 frogs, with the order of the sensory treatments randomly assigned and observers blind to the identities of the frogs.

For the rival pairs that interacted with each other under both forest and urban conditions, we selected the longest interactions, excluding the first 2 min in which the rival pair could interact to minimize carry-over effects from previous interactions. An interaction was defined as a period of at least 20 s that both males called with pauses lasting a maximum of 10 s, including the call of the male that started and ended the interaction. To reduce background noise, we filtered (> 300 and < 4,500 Hz, 24 dB roll-off) the recordings using Audacity (version 3.1.3).

Next, we characterized the selected longest interactions in terms of interaction length (sec) and temporal overlap of rival calls (% of calls), and per frog call rate (calls/min), call complexity (chucks/call) and whine amplitude measured as both root mean square (RMS) and peak-to-peak (P2P) amplitude. To calculate amplitude, we selected up to 3 nonoverlapping whines with at least 0.1 s silence before and after the call. The analyzed calls were selected from the loudest calls (based on visual inspection of oscillograms) and were never chosen from the first or last 3 calls of the interaction, because those calls typically are less loud. We calculated amplitude using *seewave* v. 2.2.0 (Sueur et al. 2008) in R v. 4.2.2 (Team R Development Core 2022), while correcting for background noise by subtracting the average RMS or P2P of the background noise 0.1 to 0.02 s before and 0.02 to 0.1 s after the call. To correct for microphone differences, we used the RMS or P2P of a nightly recorded 1 kHz calibration tone (G.R.A.S. 42AB, 114 dB). We used linear amplitude values for statistical analyses, and dB scale for reporting estimates and visualizations. Last, we excluded 4 rival pairs that had very few (< 8) or low amplitude (< 60 dB whine RMS) calls in one of their interactions from further analyses, resulting in 31 rival pairs that interacted in both forest and urban sensory conditions.

### Phonotaxis experiments

For the phonotaxis experiments (testing attraction toward sound), we selected the recordings of 15 out of the 31 rival pairs by applying additional selection criteria that had to be met during both their longest forest and urban interaction. These criteria included a minimum interaction length of 30 s and no simultaneous silence of the frogs for more than 5 s in the first or last 30 s of the interaction or twice within 30 s, to increase the likelihood that females would show phonotactic responses. Moreover, we excluded 2 rival pairs that had an interaction for more than 150 s, to minimize the number of females making a mating decision before hearing a complete interaction. Using Audacity, we removed background noise between calls, added 10 s of silence after the interactions and corrected amplitudes for microphone differences based on the RMS of the calibration tone. The final 15 rival pairs (forest rival pairs: 6, urban rival pairs: 9) consisted of 27 different males and resulted in 30 stimuli (interactions recorded in forest or urban sensory conditions). For each stimulus, we had a separate audio recording of the calls of each male of the interacting rival pair.

Phonotactic responses of female frogs to the rival interactions were assessed in a hemi-anechoic chamber (Acoustic Systems, Austin, TX, L × W × H: 2.7 × 1.8 × 1.78 m, see Fig. S2 for set-up) under both urban and forest sensory conditions (Fig. 1). Upon arrival in the lab, amplectant male–female pairs were placed in small plastic containers (9 cm ø, 5.5 cm height) with a damp paper towel on the bottom and saran wrap as a lid using low level red light. After at least 0.5 h in the lab, we transferred the pairs to one of two sound-attenuating recording boxes in which they acclimatized for 0.5 to 1 h to the first sensory treatment. Next, without using additional light, we separated individual females from their mates and placed them in the phonotaxis chamber (temperature 24 to 27 °C) beneath an acoustically permeable funnel at 90 cm distance from 2 speakers (Sherman Oaks Orb Audio Mod1, connected to Audioengine N22 amplifiers) separated by a 70° angle. Each speaker played calls from one of the males of an interacting rival pair, and we randomized which speaker would play which male per trial. Speaker volumes were calibrated daily using an artificial whine set to the P2P amplitude of the loudest whine (82 ± 0.5 dBC) at 50 cm (Voltcraft SL-100, fast, low, and max).

We exposed each female to a rival interaction for 2 min before raising the funnel and scored her choice (defined as a speaker approach to within 10 cm for at least 3 s) and decision latency (sec) using an infrared camera. We ceased the trial if a female made a choice, did not move within the first 5 min after raising the funnel, or any consecutive 2 min after her first movement, or if she did not exhibit phonotaxis within 10 min. Wall climbing or crossing the chamber back line opposite to the speakers was interpreted as a lack of motivation and scored as no choice. After 4 trials or after not making a choice twice, we returned the females to the containers with their mates using low level red light. The same acclimatization and testing procedures were repeated for the second sensory treatment. We alternated the order of the 2 sensory treatments between nights to prevent biases. In the forest and urban sensory treatments, we obtained, respectively, 152 and 140 choices made by urban females, and 90 and 80 choices made by forest females. For each phonotaxis trial, we chose the played interaction in a randomized but balanced manner regarding the rival pair and the sensory treatment during their interaction. In terms of choices for the different rival pairs, we obtained 186 choices for forest rival pairs (94 and 92 recorded in respectively forest and urban conditions), and 276 choices for urban rival pairs (140 and 136 recorded in respectively forest and urban conditions).

### Statistical analyses

We analyzed calling behavior and female preference with (generalized) linear mixed models using *lme4* v. 1.1-32 (Bates et al. 2015) in R v. 4.2.2. We assessed statistical significance of interactions between sensory treatment and frog origin and, in case of a nonsignificant interaction, their main effects using likelihood ratio tests. Alternatively, if the interaction term was found to be statistically significant, we tested treatment effects for urban and forest frogs separately using post-hoc tests to obtain estimates, standard errors, t ratios and p values using *emmeans* v. 1.8.5 (Lenth 2023). In case we found a trend in the interaction term (0.05 < *P* < 0.1), we report both main effects and post-hoc test results. All numerical covariates were standardized. We verified normality and absence of heteroskedasticity for all initial models and checked for overdispersion and zero-inflation for initial models with binomial distributions using *DHARMa* v. 0.4.6 (Hartig 2022). Graphs were created using raw data with *ggplot2* v. 3.4.1 (Wickham 2016). Complete model outputs can be found in the Supplementary Materials (Tables S3 to S6).

To assess impacts of sensory treatment on the longest rival interactions, we ran models on maximum interaction length (sec) and overlap rate, and we calculated the average of 2 rivals and their absolute differences for each call behavior trait (call rate, complexity, whine amplitude as RMS and P2P). In addition, we calculated proportional rival differences by dividing absolute rival differences by the highest value of the 2 rivals according to Weber’s law, as proportional differences might be more important for receivers than absolute differences (Weber 1948; Akre and Johnsen 2014). In all calling behavior models, we included male treatment, male origin, and their interaction as fixed effects and rival pair and male collection site as random intercept effects. All models used Gaussian distributions (identity link function), except for models on call overlap rate, that had binomial distributions (logit link function) with the number of overlapping vs. nonoverlapping calls as a response variable. To meet model assumptions, we log-transformed interaction length and sqrt-transformed absolute differences in RMS whine amplitude, call rate, and complexity and proportional differences in call rate and complexity.

To examine sensory treatment effects on mate choice, we ran models on preference strength and latency to choose (sec). Preference strength was a measure combining choices from different females. We defined preference strength (0–1) as the deviation from equal choices for both rivals, with 0 indicating equal choices for each rival (e.g. 5 out of 10 choices for both rivals) and 1 indicating all choices for one rival (e.g. 10 out of 10 choices for one rival). In case we recorded an uneven number of choices, we set equal choices between both rivals to the first possible number of choices above 50% (e.g. when recording 3 choices, 2 out of 3 choices (67%) for one rival was set as a preference strength of 0). Cases in which we only obtained one choice in an origin-treatment combination were excluded from the analyses. To run statistical models on preference strength, we combined as a response variable the number of recorded choices deviating from equal choices for both rivals and the maximum possible deviation from equal choices for both rivals using models with a binomial distribution (logit link). We analyzed whether female origin and female sensory treatment (direct effects) affected preference strength when choosing from a specific rival interaction (“stimulus”), by using preference strength collated per female origin and female treatment for each stimulus. In these models, we included female sensory treatment, female origin and their interaction as fixed effects, and stimulus as a random intercept effect. To test whether alterations in rival interactions due to sensory treatment or origin of the interacting male frogs (indirect effects) affected female preference strength, we used preference strength collated per sensory treatment for each rival pair (which had either an urban or forest origin). In these models, we included male sensory treatment, male origin and their interaction as fixed effects, and rival pair and male collection site as random intercept effects. We decided to test direct and indirect effects on preference strength in separate models because this maximized the number of data points for each treatment-origin combination. Specifically, we treated each rival interaction (regardless of the males’ origin and their sensory treatment) as a separate stimulus to test for direct effects and we did not include female origin and female sensory treatment when testing for indirect effects because our data were well balanced.

To test for direct and indirect effects of sensory treatment and frog origin on latency to choose, we used the same fixed and random effects as described above. A difference with the models on preference strength was that models on latency to choose were not collated and used latency per phonotaxis trial as response variable. Therefore, we added female ID as random intercept effect to all these models and female collection site to the latency models on the effects of female sensory treatment and female origin (direct effects). For latency models, we used a Gaussian distribution (identity link) and log-transformed the response variable to meet model assumptions.

To assess whether preference strength was higher than expected by random choice, we compared whether our measured preference strength was outside of 95% confidence intervals based on 1,000 simulations generating random choices (Table S7). Using unweighted or weighted mean preference strength (based on the number of choices per rival pair or stimulus) did not impact the results. Finally, we tested associations between mate choice and interaction characteristics, by again running models (with binomial distributions and logit links) on preference strength per stimulus. We added as fixed factors interaction length (log-transformed), overlap rate, and the proportional difference in call rate, complexity, and whine amplitude (RMS and P2P) and as random intercept effect the rival pair. We checked for multicollinearity, and all variance inflation factors were < 1.7 (Zuur et al. 2010). Next, we tested significance of associations via an information theoretic approach using *MuMIn* v. 1.47.5 (Bartoń 2023), and we report weighted averaged model estimates and associated *P* values for models < 4 AIC (small sample size corrected) from the top model (Table S8).

## Results

### Urban sensory conditions decrease maximum interaction length

**Figure 2.**
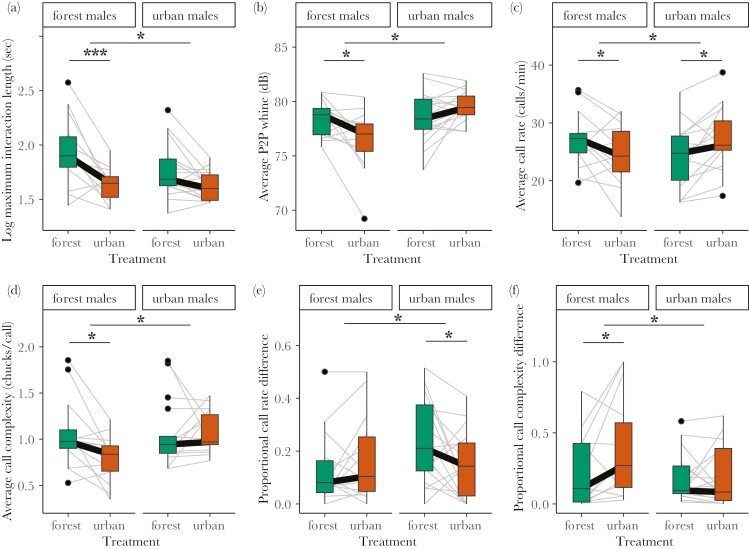
Effects of urban and forest sensory treatment on vocal interactions in urban–urban and forest–forest rival pairs. (A) Maximum interaction length (log sec). Averages of 2 rivals in a pair for (B) peak-to-peak (P2P) whine amplitude (dB), (C) call rate (calls/min), and (D) call complexity (chucks/call). Proportional differences (absolute difference divided by highest value) between 2 rivals in (E) call rate and (F) call complexity. Graphs show raw data and gray lines indicate rival pairs. Asterisks indicate statistically significance (*P < 0.05, ***P < 0.001) of interaction effects between treatment and origin (line between forest and urban frogs), and of treatment effects within origins (line within forest or urban frogs), see main text and Tables S3 to S5 for statistics.

Sensory treatment impacted maximum interaction length in an origin-dependent way (treatment × origin: *n* = 62, χ2 = 4.17, *P* = 0.041, Fig. 2A). The longest interactions between forest rivals were 51.0% shorter under urban compared to forest conditions (*n* = 28, *t* = 4.56, *P* < 0.001) and urban rivals showed a similar, but smaller, trend of 24.2% decrease under urban conditions (*n* = 34, *t* = 1.98, *P* = 0.056). How often 2 rivals overlapped their calls in these interactions did not depend on sensory treatment across or within origin (all *P* > 0.27, Table S3, Fig. S3A).

### Increased intensity in rival interactions in familiar sensory conditions

When examining the average calling behavior of rival pairs during their longest interaction, we found the effect of sensory treatment to depend on male origin for whine amplitude (treatment × origin: *n* = 62, P2P: χ^2^ = 8.60, *P* = 0.003, Fig. 2B; RMS: χ^2^ = 5.77, *P* = 0.016, Fig. S3B), call rate (χ^2^ = 10.49, *P* = 0.001, Fig. 2C), and complexity (χ^2^ = 5.34, *P* = 0.021, Fig. 2D). Forest males decreased their whine amplitude (*n* = 28, P2P: −1.6 dB, *t* = 2.68, *P* = 0.011; RMS: −1.5 dB, *t* = 2.60, *P* = 0.014), call rate (−9.6%, *t* = 2.09, *P* = 0.045), and complexity (−25.2%, *t* = 2.86, *P* = 0.008) in urban compared to forest conditions. Urban males, on the other hand, increased their call rate when exposed to urban sensory conditions (*n* = 34, + 13.2%, *t* = −2.79, *P* = 0.009), but not their amplitude or complexity (all *P* > 0.11, Table S3).

### Proportional rival differences change with sensory conditions

The absolute difference between two rivals in calling behavior during their longest interaction showed a trend in the interaction between sensory treatment and origin when looking at call rate (treatment × origin: *n* = 62, χ^2^ = 3.37, *P* = 0.07, Fig. S3E), but did not reveal an overall treatment effect (χ^2^ = 1.44, *P* = 0.23). For urban rivals, absolute differences in call rate decreased when exposed to urban sensory conditions (*n* = 34, −48.5%, *t* = 2.14, *P* = 0.04), but no pattern was found for forest rivals (*n* = 28, *t* = −0.52, *P* = 0.61). Absolute differences in other aspects of calling behavior between 2 rivals were not affected by sensory treatment (all *P* > 0.24, Table S4, Fig. S3C–F). When scaling absolute differences to the highest value of the rivals (proportional differences), we report interaction effects between sensory treatment and origin for call rate (treatment × origin: χ^2^ = 4.57, *P* = 0.033, Fig. 1E) and complexity (χ^2^ = 6.16, *P* = 0.013, Fig. 1F). Urban males decreased their proportional differences in call rate in urban compared to forest conditions (−0.11, *t* = 2.45, *P* = 0.020), while forest males increased their proportional differences in call complexity in urban compared to forest conditions (+ 0.17, *t* = −2.86, *P* = 0.007). We found no effects of sensory treatment on proportional rival differences in call amplitude (all *P* > 0.43, Table S5, Fig. S3G–H).

### Urban conditions directly affect female preference

**Figure 3.**
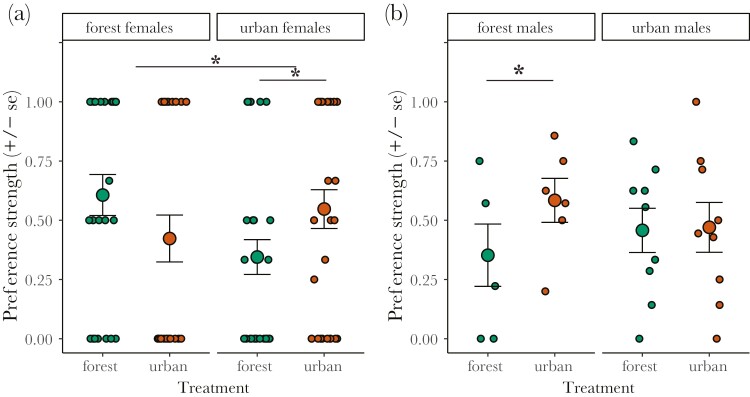
Mean (± standard error) preference strength (0 = no preference between 2 males, 1 = all choices for one male). Data are split in (A) preference of urban and forest females choosing under urban or forest sensory conditions and in (B) preference for urban–urban and forest–forest rival pairs interacting under urban or forest sensory conditions. Data points depict preference strength per stimulus (panel A) or per rival pair (panel B). Graphs show raw data. Asterisks indicate statistically significance (*P < 0.05) of interaction effects between treatment and origin (line between forest and urban frogs), and of treatment effects within origins (line within forest or urban frogs), see main text and Table S7 for statistics.

Next, we played the recorded rival interactions to urban and forest females with and without noise and light pollution to test whether their preference was affected by urban sensory conditions. The strength of female preference (i.e. how much preference deviated from equal choices for both rivals) was affected by sensory treatment in a different way for urban and forest females (treatment × origin: *n* = 462, χ^2^ = 6.66, *P* = 0.010, Fig. 3A). The urban sensory treatment increased female preference in urban females (*n* = 292, z ratio = −2.75, *P* = 0.006), but did not significantly alter preference in forest females (*n* = 170, z ratio = 1.19, *P* = 0.236). When testing whether preference strength deviated from random choices (Table S7, Fig. S5), urban females showed a preference strength that was significantly higher than random (0.55, 95% CI random choices [0.10; 0.42]) in urban conditions but not in forest conditions (0.34, [0.11; 0.40]). Forest females showed the opposite pattern, with a significantly higher preference compared to random choices under forest conditions (0.61, [0.08; 0.48]), but not under urban conditions (0.42, [0.10; 0.50]). Last, latency to choose did not differ depending on sensory treatment (all *P* > 0.63, Table S6, Fig. S4).

### Urban-induced changes in forest rival interactions impact mate choice

We tested whether alterations in rival interactions due to urban sensory conditions affect female preference strength by comparing female mate choice when listening to 2 males of a rival pair (urban–urban or forest–forest) interacting under forest versus urban conditions. We detected a trend in an interaction effect between male origin and treatment (treatment × origin: *n* = 462, χ^2^ = 2.89, *P* = 0.089, Fig. 3B). While we found no overall treatment effect (χ^2^ = 1.97, *P* = 0.16), the urban sensory treatment increased preference strength when females chose from forest rivals (*n* = 186, z ratio = −2.17, *P* = 0.030), but not when choosing from urban rivals (*n* = 276, z ratio = −0.02, *P* = 0.985). When choosing from forest rival pairs interacting under forest conditions, females did not show a statistically significant preference (0.35, random choices 95% CI: [0.00; 0.38], Table S7, Fig. S5), but when forest males interacted under urban conditions female preference was significantly higher than expected from random choices (0.58, [0.00; 0.39]). For urban rival pairs, females showed a statistically significant preference when choosing from urban rival pairs interacting under both forest (0.46, [0.03; 0.32]) and urban conditions (0.46, [0.04, 0.33]). Latency to choose did not differ depending on the sensory treatment in which the rivals interacted (all *P* > 0.19, Table S6, Fig. S4). Overall, higher preference strength was significantly associated with higher proportional call rate difference (z = 2.48, *P* = 0.01) and lower interaction length (z = 2.30, *P* = 0.02), but not any other interaction level trait (all *P* > 0.25, Table S8).

## Discussion

Research on sensory pollution has often focused on individuals or communities, but rarely on interactions between individuals (Kurvers and Hölker 2015), which is particularly important for sexual communication, as this often takes place in networks with multiple simultaneous signalers and mate choosers (McGregor and Peake 2000; Gerhardt and Huber 2002). Our aim was therefore to assess effects of urban sensory conditions on both rival interactions and mate choice in urban and forest túngara frogs. We repeatedly recorded dyadic vocal interactions of rival pairs (urban–urban males or forest–forest males), with and without noise and light pollution, allowing us to quantify emergent interaction level properties (maximum interaction length, interaction intensity, overlap rate, and rival differences), and subsequently tested female preference for the calls of the interacting males under similar sensory treatments. We found that urban sensory conditions affected both rival interactions and mate choice, with the direction of effects often depending on whether frogs were collected in urban or forest areas.

### Urban conditions affect urban and forest rival interactions differently

Maximum interaction length was significantly shorter under urban sensory conditions in forest rival pairs and showed the same trend in urban rival pairs. Since frogs are known to cease calling sooner under higher perceived predation risk (Tuttle et al. 1982), this pattern could be explained by urban noise being more disturbing and urban light being perceived as more risky because of increased visual detectability of the frogs (Rand et al. 1997). The stronger decrease in interaction length in forest rival pairs under noise and light pollution is in line with forest individuals being more vigilant (Halfwerk et al. 2019), a pattern also often reported for other taxa (Møller 2010; Uchida et al. 2019). Rival interactions can also be shortened when rivals perceive a clearer difference in the quality of their signals and therefore can settle their competition faster (Arnott and Elwood 2008), thereby reducing energetic and predation costs. This alternative explanation would suggest that the shorter interactions under urban conditions result from larger differences, as we found in forest but not urban rival pairs, or better perception of acoustic phenotypes of rivals, although we do not know of a perceptual mechanism supporting this last explanation. Furthermore, how often 2 rivals overlapped their calls was not affected by sensory treatment, which is in line with an earlier study with túngara frogs using static playbacks (Smit et al. 2022).

The intensity of the longest interaction between 2 rivals was impacted by urban sensory conditions, and, in line with our predictions, the direction of the effect depended on frog origin. Under urban sensory conditions, the forest rival pairs lowered their average call rate, amplitude and complexity, whereas the urban rival pairs increased their call rate when exposed to urban conditions. Rivalling frogs thus had more intense interactions in sensory conditions matching the conditions of their origin, indicating that exposure to unfamiliar sensory conditions could have distracted or stressed the frogs leading to more conservative or de-escalating calling strategies (Goutte et al. 2010). Urban noise and light pollution have been shown to increase conspicuousness of sexual signaling in túngara frogs when exposed to a static rival playback (Smit et al. 2022). Similarly, higher aggressiveness to rival playbacks under noisy conditions (“urban anger”) has been reported previously in white-crowned sparrows (*Zonotrichia leucophrys*) (Phillips and Derryberry 2018). These previous studies are in line with our results on urban but not on forest rival interactions, stressing the potential of population differentiation in responses to urban sensory conditions. Moreover, a translocation experiment between urban and forest sites indicated that only urban frogs show behavioral flexibility (Halfwerk et al. 2019), whereas our findings show that forest frogs also respond flexibly to sensory conditions. In forest areas, producing attractive sexual signals is balanced by higher predation and parasitic pressures (Ryan et al. 1982; Bernal et al. 2006; Akre et al. 2011), while predator and parasite abundances are much lower in urban areas or under urban sensory pollutants reducing the costs of producing conspicuous sexual signals (McMahon et al. 2017; Halfwerk et al. 2019). In terms of predation and parasitic costs, urban but not forest rival pairs thus seem to adjust their interaction intensity in an adaptive way to urban sensory pollutants, implying that urban males have adapted their behavior, via phenotypic plasticity or genetic changes, to urban sensory conditions.

In addition to the average calling behavior of a rival pair, it is insightful to examine differences between rivals’ acoustic phenotypes because larger differences should make it easier for mate choosers to discriminate and express their preference. Absolute differences generally remained under urban conditions, indicating that rivals matched their calls to the same degree (e.g. Gerhardt et al. 2000), except for a smaller difference in call rate between urban rivals when exposed to urban sensory conditions. However, proportional rival differences (absolute difference divided by the highest value) are thought to be more important than absolute differences in mate choice (Weber 1948; Akre and Johnsen 2014). Urban sensory conditions lead to lower proportional call rate differences between urban rivals, and to higher proportional call complexity differences between forest rivals, possibly because of the opposite directions of the changes in interaction intensity in urban and forest rival pairs. Changes in absolute or proportional differences can have consequences for discriminating between rivals and therefore can affect mate choice, especially when potential mates are evaluated simultaneously like in túngara frogs (Bateson and Healy 2005; Akre et al. 2011; Callander et al. 2013).

### Effects of urban sensory conditions on mate choice

When perceiving and responding to sexual signals of potential mates, mate choice can directly be influenced by human-induced changes to the environment (Candolin and Wong 2019), but the effects of urban sensory pollutants on mate choice are currently understudied (Cronin et al. 2022a). Our study sheds light on female choice between sexual signals of 2 interacting rivals, quantified as preference strength (deviation from equal choice between the 2 rivals). We show that preference strength was directly affected by urban sensory conditions but in an opposite way for urban and forest females. In line with our predictions, forest females showed a weaker (but not significantly lower) preference under urban compared to forest sensory conditions, whereas somewhat unexpectedly, urban females increased their preference when exposed to noise and light pollution. Our findings indicate that preference strength is strongest in conditions matching the females’ origin, suggesting decreased attention to or ability to discriminate between sexual signals when making a choice under unfamiliar, possibly perceived as distracting or more risky, conditions (Bonachea and Ryan 2011). While variation in latency to choose could have provided insights in potential motivational or discriminatory differences between urban and forest females under the different sensory treatments, we did not find any effects on latency. Future research should investigate mechanisms underlying differences between urban and forest female mate choice behavior.

Generally, drastic environmental changes seem to directly affect mate choice by interfering with signal perception or processing, likely decreasing benefits or increasing costs associated with mate choice. Exposure to invasive plant chemicals can, for example, decrease female preference for males with stronger immune responses in Palmate newts (*Lissotriton helveticus*) (Iglesias‐Carrasco et al. 2017), potentially lowering fitness benefits for choosing females. Similarly, exposure to anthropogenic noise can mask acoustic sexual signals and reduce female responsiveness (Huet des Aunay et al. 2014), thereby potentially increasing costs of mate searching. While there are very few studies assessing whether urban mate choosers have adapted to urban conditions, a recent study in band-legged ground crickets (*Dianemobius nigrofasciatus*) has demonstrated that urban females reared in a common garden were faster compared to forest females in localizing an acoustic sexual signal under noisy conditions (Kuriwada 2023; but see Costello and Symes 2014). As faster detection of potential mates should decrease costs associated with mate choice, such as predation or energetic costs, urban ground crickets seem to have adapted to noisy conditions. In our study, urban sensory conditions decreased preference strength only in forest females, whereas urban females showed a higher preference strength under these conditions. Even though we did not find any differences in latency to choose in our lab experiments, a higher preference strength could indicate that the choice between the 2 rivaling signalers was easier, suggesting females might have locally adapted their sensory systems to conditions of their habitat of origin (Sol et al. 2013; Harding et al. 2019).

In our female choice experiment, we also examined the indirect effects of urban sensory conditions on mate choice, namely via urban-induced changes in rival interactions, which is relatively unexplored. We found that the sensory conditions (urban or forest) in which the rivals interacted affected mate choice but only for rival pairs from forest areas. For forest rivals, females had a higher preference strength when they chose from interactions recorded under urban compared to under forest sensory conditions. The higher preference strength for forest rival interactions recorded under urban conditions is in line with our finding that higher female preference strength was associated with shorter interactions, since forest rivals significantly decreased their maximum interaction length when exposed to noise and light pollution. Rival competitions can be settled earlier when rival differences are larger, possibly making it easier for females to evaluate sexual signals. Although proportional call complexity differences were overall not associated with preference strength (but see Akre et al. 2011), we indeed we found an increase in proportional call complexity differences in forest rival pairs interacting in urban conditions. It therefore seems that changes in how 2 forest rivals interact due to light and noise pollution exposure can make it easier for females to choose between rivals, and thereby possibly benefiting choosing females. For urban rival pairs, we reported lower absolute and proportional call rate differences in interactions under urban conditions, and we found that proportional call rate difference was significantly positively associated with female preference strength. However, we did not detect changes in preference strength depending on the recording conditions of urban rival pairs, showing that for females the ability to perceive differences between 2 urban rivals was unaffected by the sensory conditions the urban rival pair interacted in. From the signaler’s perspective, a higher preference strength might indicate a more skewed distribution of matings over 2 rivals, and thereby stronger selection pressures on sexual signaling. Overall, we demonstrate that changes in how rivals interact when exposed to urban sensory conditions can indirectly affect mate choice, which, despite its potential fitness consequences, has rarely been studied previously.

This study focused on dyadic rival interactions with one female frog choosing at the time, the simplest form of a communication network. Communication networks can, however, consist out of many more signalers and mate choosers, resulting in complex chorus dynamics impacting female preference (Greenfield and Rand 2000; Calsbeek et al. 2022; Larter and Ryan 2024). Moreover, sensory conditions can affect communication networks more indirectly through changes in, for example, community composition, spatial distribution and species interactions (Francis et al. 2009; Naguib 2013). Our study can form a basis for understanding the effects of urbanization on sexual communication in interactive settings, and further work could build upon this by examining more complex communication networks.

## Conclusion

By examining dyadic rival interactions and letting females choose from playbacks of these interactions, we studied the effects of urbanization on a communication network in which sexual signaling as well as mate choice could be shaped by the phenotypes of conspecifics. Our study demonstrated that noise and light pollution can impact both signalers and, directly as well as indirectly, mate choosers. Interestingly, the effects of urban sensory pollution were often origin-dependent, with urban and forest frogs showing opposite responses. When tested in sensory conditions matching the frogs’ origin, we found that rival interactions were most intense and preference strength was highest, suggesting that urban frogs might have adapted their sexual communication behavior, via plasticity and/or genetic processes, to urbanization.

## Supplementary Material

arae088_suppl_Supplementary_Material

## Data Availability

Analyses reported in this article can be reproduced using the data provided by Smit et al. (2024).
