# Supplementary material for: Urban sensory conditions alter rival interactions and mate choice in urban and forest túngara frogs
Source: Behav Ecol. 2024 Oct 26;35(6):arae088. doi: 10.1093/beheco/arae088 (PMC11558454; doi:10.1093/beheco/arae088)
Supplement: arae088_suppl_Supplementary_Material [file arae088_suppl_supplementary_material.docx]

Supplementary material

Figure S1 Map showing the locations of the 6 urban and 4 forest sites (see Tbl. S1 for coordinates and description).


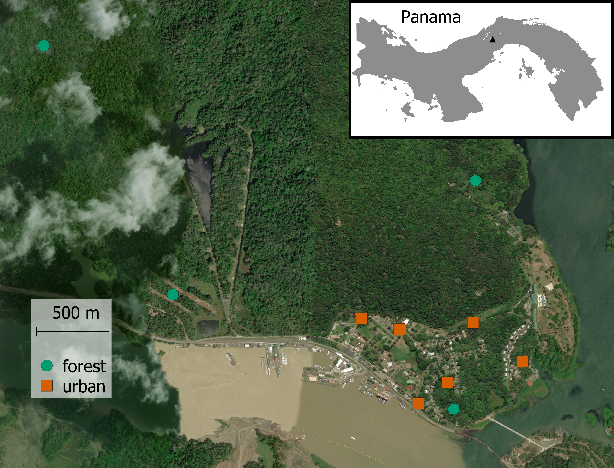


Table S1 Overview of urban and forest frog collection sites. Mean and standard deviation of night-time light levels per site are based on five male calling locations using a lux meter (HT Instruments HT309) pointed upwards. The number of collected males, the number of recorded interacting males and the subset used as phonotaxis stimuli are depicted per collection site. Similarly, the number of collected amplexed pairs, unique number of collected females and number of females that made one or more choices during the phonotaxis experiment are indicated per collection site.

| Site | Coordinates (EPSG:4326 - WGS84 CRS) | Mean±sd light level (lux) | Collected males | Rival interaction males (phonotaxis stimuli) | Collected pairs (unique females) | Choosing females |
| --- | --- | --- | --- | --- | --- | --- |
| *Urban collection sites* | | | | | | |
| Stairs | 9.115101,  -79.699862 | 1.97±1.69 | 8 | 4 (3) | 12 (11) | 10 |
| Santa Cruz | 9.120339,  -79.703418 | 1.57±1.40 | 16 | 7 (3) | 11 (10) | 9 |
| Insectaries | 9.119689,  -79.701056 | 2.96±4.81 | 16 | 5 (5) | 19 (19) | 19 |
| Building 183 | 9.116392,  -79.698036 | 0.11±0.21 | 8 | 2 (0) | 3 (3) | 2 |
| Kent’s Marsh | 9.120090,  -79.696427 | 0.20±0.39 | 8 | 2 (0) | 1 (1) | 1 |
| Marina | 9.117693,  -79.693297 | 0.85±1.08 | 16 | 4 (4) | 9 (9) | 8 |
| *Forest collection sites* | | | | | | |
| Pipeline Bridge | 9.137279,  -79.723399 | 0.00±0.00 | 16 | 6 (5) | 1 (1) | 0 |
| Pipeline Start | 9.121832,  -79.715290 | 0.00±0.00 | 8 | 3 (2) | 7 (6) | 4 |
| Ditch | 9.114733,  -79.697640 | 0.00±0.00 | 8 | 3 (2) | 12 (12) | 12 |
| La Chunga | 9.128899,  -79.696274 | 0.00±0.00 | 16 | 5 (2) | 15 (15) | 15 |

Figure S2 The set-up in the phonotaxis chamber on scale. L1-L4 = light 1-4; NS1-NS2 = noise speaker 1-2; F = funnel; SS1-SS2 = stimulus speaker 1-2; CZ1-CZ2 = choice zone of stimulus speaker 1-2; red dot 1-11 = calibration point 1-11. SS1 and SS2 were placed on the ground directed towards F, L1 and L2 were positioned on the ceiling in an angle so that their maximum light levels were right under NS1 and F and NS1, NS2, L3 and L4 were hanging on the ceiling facing downwards.

***
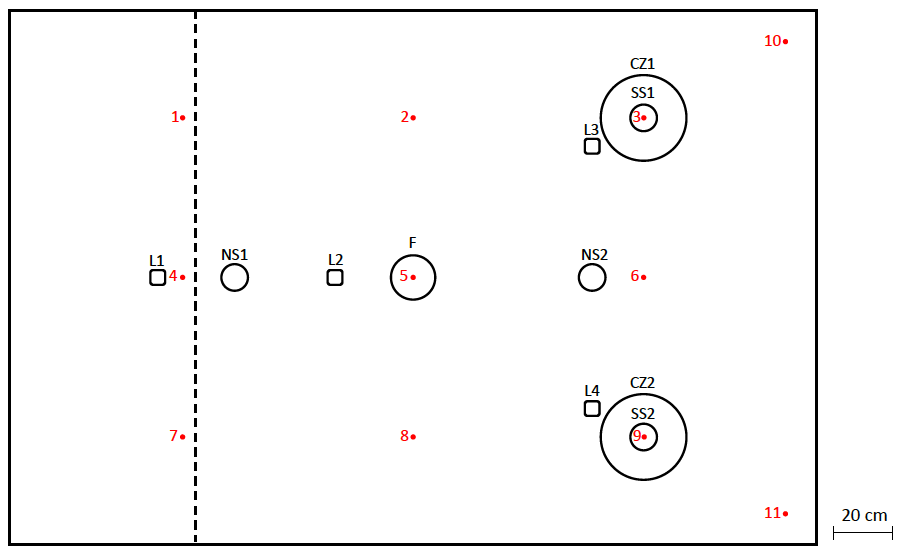
***

Table S2 Light and sound levels of the urban and forest light treatment, and of the chorus used to calibrate the noise levels at 11 calibration points in the phonotaxis chamber (see Fig. S2) measured on ground level with respectively HT instruments HT309 and Voltcraft SL-100 (A-weighted, fast, low, and max).

| **Calibration Point** | 1 | 2 | 3 | 4 | 5 | 6 | 7 | 8 | 9 | 10 | 11 |
| --- | --- | --- | --- | --- | --- | --- | --- | --- | --- | --- | --- |
| **Light level urban (lx)** | 0.07 | 0.15 | 1.08 | 0.98 | 1.95 | 0.27 | 0.07 | 0.11 | 1.16 | 0.00 | 0.00 |
| **Light level forest (lx)** | 0.00 | 0.00 | 0.00 | 0.00 | 0.00 | 0.00 | 0.00 | 0.00 | 0.00 | 0.00 | 0.00 |
| **Chorus (dBA)** | 69.0 | 71.7 | 68.6 | 71.1 | 73.7 | 71.1 | 68.2 | 71.9 | 69.1 | 65.9 | 65.7 |

Table S3 Results on the effects of male treatment and male origin on interaction level traits (maximum interaction length, overlap rate, whine amplitude (P2P and RMS), call rate and complexity). Initial models (GLMM) are specified below. χ² values and p values were obtained by likelihood ratio tests of models with and without the factor of interest, starting with the interaction term, and estimates and standard errors were obtained from the summary table of the model. In case of a non-significant interaction term, we subsequently removed the interaction term and report analyses testing for main effects of sensory treatment and frog origin. Alternatively, if the interaction team was found to be statistically significant, we tested treatment effects for urban and forest frogs using post-hoc tests to obtain estimates, standard errors, t ratios and p values (bold values indicated p values < 0.05). In case we found a trend in the interaction term (0.05 < p < 0.1), we report both main effects and post-hoc test results. Estimates for ‘treatment_male’ and ‘origin_male’ indicate the effects of urban compared to forest treatment or origin, estimates are not back transformed.

| **Model** | **Explanatory** | **Estimate** | **s.e.** | | **χ² or t ratio** | | **P** |
| --- | --- | --- | --- | --- | --- | --- | --- |
| Log10 (Interaction length) ~ treatment_male*origin_male + (1\|rival_pair) + (1\|site_male),  family = Gaussian, link = identity | | | | | | | |
| *Combined*  *(interaction)* | Intercept | 1.94321 | 0.05980 | |  | |  |
|  | Treatment_male | -0.30892 | 0.06780 | |  | |  |
|  | Origin_male | -0.18864 | 0.08075 | |  | |  |
|  | Interaction | 0.18698 | 0.09156 | | 0.04126 | | **0.04126** |
| *Forest frogs* | Intercept | 1.943205 | 0.0632 | |  | |  |
|  | Treatment_male | -0.309 | 0.0678 | | 4.556 | | **0.0001** |
| *Urban frogs* | Intercept | 1.754568 | 0.0578 | |  | |  |
|  | Treatment_male | -0.122 | 0.0615 | | 1.982 | | 0.0570 |
| cbind(overlaps, total_calls - overlaps) ~ treatment_male*origin_male + (1\| rival_pair ) + (1\|site_male), family = "binomial", link = logit | | | | | | | |
| *Combined*  *(interaction)* | Intercept | -5.18588 | 0.44708 | |  | |  |
|  | Treatment_male | 0.07422 | 0.61120 | |  | |  |
|  | Origin_male | 0.20547 | 0.60406 | |  | |  |
|  | Interaction | 0.49297 | 0.76317 | | 0.4289 | | 0.5125 |
| *Combined  (no interaction)* | Intercept | -5.2873 | 0.4271 | |  | |  |
|  | Treatment_male | 0.3857 | 0.3567 | | 1.15329 | | 0.2829 |
|  | Origin_male | 0.4154 | 0.5057 | | 0.64438 | | 0.4221 |
| P2P_whine (linear) ~ treatment_male*origin_male + (1\|rival_pair) + (1\|site_male),  family = Gaussian, link = identity | | | | | | | |
| *Combined*  *(interaction)* | Intercept | 0.0171709 | 0.0011679 | |  | |  |
|  | Treatment_male | -0.0028755 | 0.0010735 | |  | |  |
|  | Origin_male | 0.0008012 | 0.0015665 | |  | |  |
|  | Interaction | 0.0044139 | 0.0014497 | | 8.5989 | | **0.003364** |
| *Forest frogs* | Intercept | 0.0172 | 0.00119 | |  | |  |
|  | Treatment_male | -0.00288 | 0.001074 | | 2.679 | | **0.0114** |
| *Urban frogs* | Intercept | 0.0181 | 0.00108 | |  | |  |
|  | Treatment_male | 0.00154 | 0.000974 | | -1.579 | | 0.1238 |
| RMS_whine (linear) ~ treatment_male*origin_male + (1\|rival_pair) + (1\|site_male),  family = Gaussian, link = identity | | | | | | | |
| *Combined*  *(interaction)* | Intercept | 0.0072904 | 0.0007024 | |  | |  |
|  | Treatment_male | -0.0011608 | 0.0004466 | |  | |  |
|  | Origin_male | 0.0014642 | 0.0009367 | |  | |  |
|  | Interaction | 0.0014691 | 0.0006031 | | 5.7704 | | **0.0163** |
| *Forest frogs* | Intercept | 0.00729 | 0.000709 | |  | |  |
|  | Treatment_male | -0.001161 | 0.000447 | | 2.599 | | **0.0146** |
| *Urban frogs* | Intercept | 0.00875 | 0.000634 | |  | |  |
|  | Treatment_male | 0.000308 | 0.000405 | | -0.761 | | 0.4529 |
| Call_rate ~ treatment_male*origin_male + (1\|rival_pair) + (1\|site_male),  family = Gaussian, link = identity | | | | | | | |
| *Combined*  *(interaction)* | Intercept | 26.776 | | 1.944 | |  |  |
|  | Treatment_male | -2.581 | | 1.237 | |  |  |
|  | Origin_male | -3.152 | | 2.588 | |  |  |
|  | Interaction | 5.708 | | 1.671 | | 10.486 | **0.001203** |
| *Forest frogs* | Intercept | 26.8 | | 1.96 | |  |  |
|  | Treatment_male | -2.58 | | 1.24 | | 2.086 | **0.0447** |
| *Urban frogs* | Intercept | 23.7 | | 1.74 | |  |  |
|  | Treatment_male | 3.13 | | 1.12 | | -2.785 | **0.0088** |
| Call_complexity ~ treatment_male*origin_male + (1\|rival_pair) + (1\|site_male),  family = Gaussian, link = identity | | | | | | | |
| *Combined*  *(interaction)* | Intercept | 1.059539 | | 0.100859 | |  |  |
|  | Treatment_male | -0.266736 | | 0.093155 | |  |  |
|  | Origin_male | -0.007737 | | 0.135078 | |  |  |
|  | Interaction | 0.293631 | | 0.125795 | | 5.3373 | **0.02087** |
| *Forest frogs* | Intercept | 1.060 | | 0.1021 | |  |  |
|  | Treatment_male | -0.2667 | | 0.0932 | | 2.863 | **0.0077** |
| *Urban frogs* | Intercept | 1.052 | | 0.0922 | |  |  |
|  | Treatment_male | 0.0269 | | 0.0845 | | -0.318 | 0.7527 |

Table S4 Results on the effects of male treatment and male origin on absolute rival differences (whine amplitude (P2P and RMS), call rate and complexity). See Tbl. S3 for details.

| **Model** | **Explanatory** | **Estimate** | **s.e.** | **χ² or t ratio** | **P** |
| --- | --- | --- | --- | --- | --- |
| Absolute difference P2P_whine (linear) ~ treatment_male*origin_male + (1\|rival_pair) + (1\|site_male),  family = Gaussian, link = identity | | | | | |
| *Combined*  *(interaction)* | Intercept | 0.006586 | 0.001068 |  |  |
|  | Treatment_male | -0.000569 | 0.001071 |  |  |
|  | Origin_male | 0.002401 | 0.001442 |  |  |
|  | Interaction | -0.00009097 | 0.001447 | 0.0042268 | 0.9482 |
| *Combined  (no interaction)* | Intercept | 0.006611 | 0.000989 |  |  |
|  | Treatment_male | -0.000619 | 0.000708 | 0.78 | 0.377 |
|  | Origin_male | 0.002355 | 0.001247 | 3.60 | 0.058 |
| Absolute difference sqrt(RMS_whine (linear)) ~ treatment_male*origin_male + (1\|rival_pair) + (1\|site_male),  family = Gaussian, link = identity | | | | | |
| *Combined*  *(interaction)* | Intercept | 0.04169 | 0.00786 |  |  |
|  | Treatment_male | 0.00375 | 0.00611 |  |  |
|  | Origin_male | 0.02567 | 0.01053 |  |  |
|  | Interaction | -0.00499 | 0.00825 | 0.388 | 0.53 |
| *Combined  (no interaction)* | Intercept | 0.04306 | 0.00752 |  |  |
|  | Treatment_male | 0.00101 | 0.00406 | 0.06 | 0.80 |
|  | Origin_male | 0.02318 | 0.00969 | 5.38 | **0.02** |
| Absolute difference call_rate ~ treatment_male*origin_male + (1\|rival_pair) + (1\|site_male),  family = Gaussian, link = identity | | | | | |
| *Combined*  *(interaction)* | Intercept | 1.710 | 0.299 |  |  |
|  | Treatment_male | 0.180 | 0.346 |  |  |
|  | Origin_male | 0.654 | 0.404 |  |  |
|  | Interaction | -0.853 | 0.467 | 3.37 | 0.067 |
| *Combined  (no interaction)* | Intercept | 1.944 | 0.272 |  |  |
|  | Treatment_male | -0.288 | 0.241 | 1.435 | 0.23 |
|  | Origin_male | 0.228 | 0.329 | 0.585 | 0.44 |
| *Forest frogs* | Intercept | 1.70 | 0.314 |  |  |
|  | Treatment_male | 0.180 | 0.346 | -0.519 | 0.6072 |
| *Urban frogs* | Intercept | 2.37 | 0.287 |  |  |
|  | Treatment_male | -0.673 | 0.314 | 2.142 | **0.0396** |
| Absolute difference sqrt(call_complexity) ~ treatment_male*origin_male + (1\|rival_pair) + (1\|site_male),  family = Gaussian, link = identity | | | | | |
| *Combined*  *(interaction)* | Intercept | 0.4076 | 0.0857 |  |  |
|  | Treatment_male | 0.1326 | 0.0794 |  |  |
|  | Origin_male | -0.0032 | 0.1155 |  |  |
|  | Interaction | -0.1293 | 0.1072 | 1.52 | 0.22 |
| *Combined  (no interaction)* | Intercept | 0.4431 | 0.0806 |  |  |
|  | Treatment_male | 0.0617 | 0.0537 | 1.333 | 0.25 |
|  | Origin_male | -0.0679 | 0.1023 | 0.319 | 0.57 |

Table S5 Results on the effects of male treatment and male origin on proportional rival differences (whine amplitude (P2P and RMS), call rate and complexity). See Tbl. S3 for details.

| **Model** | **Explanatory** | **Estimate** | **s.e.** | **χ² or t ratio** | **P** |
| --- | --- | --- | --- | --- | --- |
| Proportional difference P2P_whine (linear) ~ treatment_male*origin_male + (1\|rival_pair) + (1\|site_male),  family = Gaussian, link = identity | | | | | |
| *Combined*  *(interaction)* | Intercept | 0.3136 | 0.0432 |  |  |
|  | Treatment_male | 0.0271 | 0.0400 |  |  |
|  | Origin_male | 0.0857 | 0.0583 |  |  |
|  | Interaction | -0.0878 | 0.0540 | 2.7 | 0.1 |
| *Combined  (no interaction)* | Intercept | 0.3376 | 0.0407 |  |  |
|  | Treatment_male | -0.0210 | 0.0276 | 0.592 | 0.44 |
|  | Origin_male | 0.0418 | 0.0517 | 0.692 | 0.41 |
| Proportional difference RMS_whine (linear) ~ treatment_male*origin_male + (1\|rival_pair) + (1\|site_male),  family = Gaussian, link = identity | | | | | |
| *Combined*  *(interaction)* | Intercept | 0.2526 | 0.0594 |  |  |
|  | Treatment_male | 0.0609 | 0.0492 |  |  |
|  | Origin_male | 0.1795 | 0.0800 |  |  |
|  | Interaction | -0.0980 | 0.0664 | 2.24 | 0.13 |
| *Combined  (no interaction)* | Intercept | 0.27951 | 0.05658 |  |  |
|  | Treatment_male | 0.00719 | 0.03370 | 0.05 | 0.828 |
|  | Origin_male | 0.13053 | 0.07273 | 3.40 | 0.065 |
| Proportional difference call_rate ~ treatment_male*origin_male + (1\|rival_pair) + (1\|site_male),  family = Gaussian, link = identity | | | | | |
| *Combined*  *(interaction)* | Intercept | 0.3303 | 0.0584 |  |  |
|  | Treatment_male | 0.0427 | 0.0636 |  |  |
|  | Origin_male | 0.1171 | 0.0786 |  |  |
|  | Interaction | -0.1842 | 0.0858 | 4.57 | **0.033** |
| *Forest frogs* | Intercept | 0.323 | 0.0602 |  |  |
|  | Treatment_male | 0.0427 | 0.0636 | -0.672 | 0.5064 |
| *Urban frogs* | Intercept | 0.452 | 0.0550 |  |  |
|  | Treatment_male | -0.1415 | 0.0577 | 2.454 | **0.0196** |
| Proportional difference call_complexity ~ treatment_male*origin_male + (1\|rival_pair) + (1\|site_male),  family = Gaussian, link = identity | | | | | |
| *Combined*  *(interaction)* | Intercept | 0.38116 | 0.07406 |  |  |
|  | Treatment_male | 0.17807 | 0.06237 |  |  |
|  | Origin_male | -0.00579 | 0.09978 |  |  |
|  | Interaction | -0.21257 | 0.08422 | 6.16 | **0.013** |
| *Forest frogs* | Intercept | 0.381 | 0.0771 |  |  |
|  | Treatment_male | 0.1781 | 0.0624 | -2.855 | **0.0079** |
| *Urban frogs* | Intercept | 0.375 | 0.0706 |  |  |
|  | Treatment_male | -0.0345 | 0.0566 | 0.610 | 0.5469 |

Figure S3 Effects of urban and forest sensory treatment on vocal interactions in urban-urban and forest-forest rival pairs. A) overlap rate (%), B) averages of rival pair in RMS whine amplitude (dB), absolute differences in C) RMS whine amplitude (dB), D) P2P whine amplitude, E) call rate and F) call complexity, proportional differences (absolute difference divided by highest value) between two rivals in G) RMS whine amplitude and H) P2P whine amplitude. Graphs show raw data, grey lines indicate rival pairs. Asterisks indicate statistically significance ﻿(* P < 0.05) of interaction effects between treatment and origin (line between forest and urban frogs), and of treatment effects within origins (line within forest or urban frogs), see main text and Tbl. S3-5 for statistics.

Table S6 Results on the effects of female/male treatment and female/male origin on latency to choose (seconds) and preference strength (0-1). See Tbl. S3 for details on statistics.

| **Model** | **Explanatory** | **Estimate** | **s.e.** | **χ² or t ratio** | **P** |
| --- | --- | --- | --- | --- | --- |
| Log10 (latency) ~ treatment_female*origin_female + (1\|female_ID) + (1\|stimulus) + (1\|site_female), family = Gaussian, link = identity | | | | | |
| *Combined*  *(interaction)* | Intercept | 1.8103 | 0.0465 |  |  |
|  | Treatment_female | -0.0234 | 0.0497 |  |  |
|  | Origin_female | -0.0275 | 0.0582 |  |  |
|  | Interaction | 0.0120 | 0.0625 | 0.0374 | 0.85 |
| *Combined  (no interaction)* | Intercept | 1.8069 | 0.0429 |  |  |
|  | Treatment_female | -0.0158 | 0.0299 | 0.288 | 0.59 |
|  | Origin_female | -0.0220 | 0.0506 | 0.187 | 0.67 |
| Log10 (latency) ~ treatment_male*origin_male + (1\|female_ID) + (1\|rival_pair) + (1\|site_male),  family = Gaussian, link = identity | | | | | |
| *Combined*  *(interaction)* | Intercept | 1.7739 | 0.0487 |  |  |
|  | Treatment_male | 0.0226 | 0.0477 |  |  |
|  | Origin_male | -0.0235 | 0.0601 |  |  |
|  | Interaction | 0.0273 | 0.0615 | 0.196 | 0.66 |
| *Combined  (no interaction)* | Intercept | 1.7660 | 0.0453 |  |  |
|  | Treatment_male | 0.0389 | 0.0302 | 1.655 | 0.20 |
|  | Origin_male | -0.0102 | 0.0521 | 0.038 | 0.84 |
| cbind(choices deviating from equal choices, choices not deviating from equal choices) ~ treatment_female * origin_female + (1\|stimulus), family = binomial, link= logit | | | | | |
| *Combined*  *(interaction)* | Intercept | 0.220 | 0.431 |  |  |
|  | Treatment_female | -0.666 | 0.562 |  |  |
|  | Origin_female | -1.166 | 0.486 |  |  |
|  | Interaction | 1.820 | 0.719 | 6.66 | **0.0099** |
| *Forest frogs* | Intercept | 0.220 | 0.431 |  |  |
|  | Treatment_female | -0.666 | 0.562 | 1.186 | 0.2358 |
| *Urban frogs* | Intercept | -0.946 | 0.363 |  |  |
|  | Treatment_female | 1.154 | 0.420 | -2.745 | **0.0061** |
| cbind(choices deviating from equal choices, choices not deviating from equal choices) ~ treatment_male * origin_male + (1\|rival_pair) + (1\|site_male), family = binomial, link= logit | | | | | |
| *Combined*  *(interaction)* | Intercept | -0.542 | 0.570 |  |  |
|  | Treatment_male | 1.028 | 0.474 |  |  |
|  | Origin_male | 0.415 | 0.756 |  |  |
|  | Interaction | -1.020 | 0.603 | 2.89 | 0.089 |
| *Combined  (no interaction)* | Intercept | -0.2311 | 0.5241 |  |  |
|  | Treatment_male | 0.4071 | 0.2899 | 1.972 | 0.16 |
|  | Origin_male | -0.0974 | 0.6733 | 0.021 | 0.88 |
| *Forest frogs* | Intercept | -0.542 | 0.570 |  |  |
|  | Treatment_male | 1.0276 | 0.474 | -2.166 | **0.0303** |
| *Urban frogs* | Intercept | -0.127 | 0.500 |  |  |
|  | Treatment_male | 0.0072 | 0.373 | -0.019 | 0.9845 |

Figure S4 Latency to choose (log seconds) split in A) urban and forest females choosing under urban or forest sensory conditions and split in B) females choosing for urban and forest rival pairs interacting under urban or forest sensory conditions. Graph depicts raw data. See main text and Tbl. S6 for statistics.

Table S7 Preference strength (0-1) obtained in the phonotaxis experiments and 95% confidence interval (CI) based on 1000 simulations of random choices. Both unweighted and weighted (based on number of choices per stimulus or rival pairs) are reported. Data is split in female origin and female sensory treatment, or in male origin and male sensory treatment.

| **Origin** | **Sensory treatment** | **Measured  female preference (weighted)** | **Unweighted simulated preference strength (95% CI)** | **Weighted simulated preference strength (95% CI)** |
| --- | --- | --- | --- | --- |
| **Female origin and sensory treatment** | | | | |
| *Forest females* | Forest | 0.61 (0.55) | 0.08; 0.48 | 0.08; 0.45 |
|  | Urban | 0.42 (0.42) | 0.10; 0.50 | 0.10; 0.48 |
| *Urban females* | Forest | 0.34 (0.32) | 0.11; 0.40 | 0.11; 0.39 |
|  | Urban | 0.55 (0.53) | 0.10; 0.42 | 0.11; 0.40 |
| **Male origin and sensory treatment** | | | | |
| *Forest males* | Forest | 0.35 (0.35) | 0.00; 0.38 | 0.00; 0.39 |
|  | Urban | 0.58 (0.57) | 0.00; 0.39 | 0.00; 0.38 |
| *Urban males* | Forest | 0.46 (0.46) | 0.03; 0.32 | 0.03; 0.32 |
|  | Urban | 0.47 (0.46) | 0.04; 0.33 | 0.04; 0.33 |

Figure S5 Preference strength (0-1) split in A) preference of urban and forest females choosing under urban or forest sensory conditions and split in B) preference for urban and forest rival pairs interacting under urban or forest sensory conditions. Open circles indicate preference strength obtained in the phonotaxis experiments and the error bars indicated 95% confidence intervals based on 1000 simulations. Mean preference strength was not weighted depending on the number of choices per stimulus or rival pair. See main text and Tbl. S7 for statistics.

******

Table S8 Results on the association between preference strength and interaction level characteristics (proportional call rate difference, interaction length, proportional call complexity difference, overlap rate and proportional P2P whine amplitude difference). Table shows the result of weighted model averaging (delta < 4). See main text for details on statistics.

| **Explanatory** | **Estimate** | **s.e.** | **z value** | **P** |
| --- | --- | --- | --- | --- |
| cbind(choices deviating from equal choices, choices not deviating from equal choices) ~ prop_diff_call_rate + log10(interaction length) + prop_diff_complexity + overlap rate + prop_diff_P2P, (1\|rival_pair), family = binomial, link= logit | | | | |
| Intercept | -0.16508011 | 0.2524651 |  |  |
| prop_diff_call_rate | 0.57309120 | 0.2201351 | 2.4803655 | **0.0131248** |
| log10(interaction length) | -0.51587032 | 0.2141835 | 2.2951737 | **0.0217232** |
| prop_diff_complexity | 0.23178010 | 0.1948456 | 1.1320449 | 0.2576156 |
| overlap rate | 0.14072933 | 0.2309802 | 0.5798130 | 0.5620407 |
| prop_diff_P2P | -0.06987971 | 0.2106951 | 0.3156274 | 0.7522853 |
